# What do Indian children drink when they do not receive water? Statistical analysis of water and alternative beverage consumption from the 2005–2006 Indian National Family Health Survey

**DOI:** 10.1186/s12889-015-1946-4

**Published:** 2015-07-05

**Authors:** Jasmine Fledderjohann, Pat Doyle, Oona Campbell, Shah Ebrahim, Sanjay Basu, David Stuckler

**Affiliations:** Department of Sociology, University of Oxford, Oxford, UK; Department of Non-Communicable Disease Epidemiology, London School of Hygiene & Tropical Medicine, London, UK; South Asia Chronic Disease Network, Public Health Foundation of India, Delhi, India; Stanford University School of Medicine, Stanford University, Stanford, CA USA

## Abstract

**Background:**

Over 1.2 billion people lack access to clean water. However, little is known about what children drink when there is no clean water. We investigated the prevalence of receiving no water and what Indian children drink instead.

**Methods:**

We analysed children’s beverage consumption using representative data from India’s National Family and Health Survey (NFHS-3, 2005–2006). Consumption was based on mothers’ reports (*n* = 22,668) for children aged 6–59 months (*n* = 30,656).

**Results:**

About 10 % of Indian children had no water in the last 24 h, corresponding to 12,700,000 children nationally, (95 % CI: 12,260,000 to 13,200,000). Among children who received no water, 23 % received breast or fresh milk and 24 % consumed formula, “other liquid”, juice, or two or more beverages. Children over 2 were more likely to consume non-milk beverages, including tea, coffee, and juice than those under 2 years. Those in the lowest two wealth quintiles were 16 % less likely to have received water (OR = 0.84; 95 % CI: 0.74 to 0.96). Compared to those living in households with bottled, piped, or tanker water, children were significantly less likely to receive water in households using well water (OR = 0.75; 95 % CI: 0.64 to 0.89) or river, spring, or rain water (OR = 0.70; 95 % CI: 0.53 to 0.92) in the last 24 h.

**Conclusions:**

About 13 million Indian children aged 6–59 months received no water in the last 24 h. Further research is needed to assess the risks potentially arising from insufficient water, caffeinated beverages, and high sugar drinks at early stages of life.

**Electronic supplementary material:**

The online version of this article (doi:10.1186/s12889-015-1946-4) contains supplementary material, which is available to authorized users.

## Background

What do children drink when they do not receive water?

An estimated 768 million people lack access to clean drinking water worldwide [1, 2]. About 144 million of these persons are in India alone [3], where one-quarter of the population has no drinking water available on their premises [4]. Even in Kerala, one of India’s relatively developed states, about 70 % of households lack clean tap water [5]. A 2011 government survey of 12 of India’s biggest states found that more than half of India’s cities have no piped water systems, and, of those with piped water systems, four fifths had water access for fewer than five hours per day [6, 7]. The consequences of unclean water are well-established; unclean water poses significant risks of diarrhoea, opportunistic infections, and consequent malnutrition, especially to immunologically vulnerable groups, including children [8–11]. Thus mothers may be advised to avoid providing children with water from sources that may be contaminated [12].

Relatively less studied, yet also potentially hazardous, are the consequences for health when children receive little or no water altogether. First, dehydration poses a serious health risk. Clinical studies find it can lead to multiple adverse outcomes, including postnatal weight loss [13], diminished cognitive performance [14–16], and, in extreme cases, death [13, 17, 18]. While there is no universal minimum water requirement, as hydration needs depend on the levels of water lost, the WHO estimates of 7.5 l per capita per day as a bare minimum for survival, with approximately 2.5 l for drinking, 2 l for basic hygiene, and 3 l for cooking [19]. To fulfil human needs for bathing, food preparation, handwashing, and toilet facilities combined, 50 l per person per day are needed for adults [20] and “two buckets” (about 20 l) for children [21]. WHO and UNICEF recommend exclusively breastfeeding children for the first 6 months of life, followed by the introduction of complementary foods, including water. Hot climates and inadequate sanitation, both prevalent risk factors in India, are likely to increase substantially the level of water needed for children’s survival by increasing the amount of water lost to evaporation from the skin (i.e. sweating) and diarrheal wastage arising from sanitation-related infections [20, 22, 23].

Second, there are concerns that, particularly in settings where water is unclean or scarce, children may drink sugar-sweetened beverages, such as juices and fizzy drinks, or caffeine, such as tea and coffees, also often containing high added sugar. Given the health risks associated with dehydration [24], intake of sugar-sweetened beverages may be preferable to consuming no fluids at all in the short-term; however, in the long-term, excessive sugar consumption has been tied to risk of non-communicable diseases such as obesity and diabetes [25, 26]. While it is unclear exactly to what extent individuals with limited access to clean water are at risk for non-communicable diseases such as diabetes and obesity, India’s ongoing nutrition transition and rising burden of chronic conditions points to the growing importance of adherence to WHO sugar consumption guidelines [27]. Anecdotal reports have suggested that children living in slums are fed sugar-sweetened beverages in bottles in Mexico, Brazil, India, and other deprived settings [28]. These sugar-sweetened beverages may pose risks of obesity and early-onset diabetes, as well as cardiovascular diseases in adult life [26, 29].

Here, we examine the prevalence of children who receive no water in India. We further investigate what children drink when they are reported to have no water, using nationally representative survey data covering Indian mothers and children, from the latest available National Family and Health Survey in years 2005–2006.

## Methods

We analysed data from India’s National Family and Health Survey (NFHS-3), collected by 18 research organizations under the direction of the Indian Ministry of Health and Family Welfare between November 2005 and August 2006 [30]. The data are publicly available and free of charge from the Demographic and Health Surveys website [31]. A probability proportional to size sampling method was selected, with a two-stage design (villages, households) in rural areas and a three-stage design (wards, Census enumeration blocks, households) in urban areas. A stratified sampling method was used in the first stage to ensure representativeness of the sample on the basis of village size, primary labour modality, caste, female literacy, HIV prevalence, and a variety of other indicators [32].

The sample, which is representative at both the national and state level, includes 124,385 women aged 15–49 and 74,369 men aged 15–54 across each of India’s 29 states. Our analytic sample was limited to regular female residents of the household (interviewed visitors were excluded). Population weights, which adjust for the sampling design and nonresponse (response rate = 93.5 %), were applied to all analyses using the weighting variable created by the NFHS survey team [33]. Less than 5 % of eligible women were not sampled due to not being home (2.9 %), postponing the interview (.1 %), refusing to participate (1.5 %), being incapacitated (.3 %), or some other reason (.3 %). Data on food and water consumption were collected only for living children born within the five year period preceding the interview. Since introduction of water into the diet is not recommended for children under 6 months of age, we restricted the sample to living children aged 6–59 months (*n* = 30,656) Additional file 1.

In the first stage of the analysis, we evaluated the prevalence of children who received no water using a synthetic cohort—that is, examining water consumption patterns within each 1 month age group. Water consumption was measured based on the mother’s report of children’s consumption from a questionnaire covering the preceding 24 h for 24 food items. For children under the age of 5 years, mothers were asked: “Now I would like to ask you about liquids (NAME) drank yesterday during the day or at night. Did (NAME) drink:” The list included plain water, fruit juice, tea or coffee, tinned, powdered, or fresh milk, commercially produced formula, and other liquids, in addition to a variety of solid foods. Children whose mothers reported “No” to plain water were coded as not consuming water; children whose mothers reported “No” to all beverage questions were reported as not consuming any beverages. An additional question asked how many times the child was breastfed in the preceding 24 h; children who were reported as having been breastfed 0 times during that period were coded as not having breast milk in the last 24 h. Missing data did not exceed 4 % for most food intake variables in the analysis, but was higher for water consumption (23.1 %, *n* = 10,516). To estimate the population-level prevalence rates, we used UN population figures for 2005 which indicated a total population of 6–59 month old Indian children of 148,698,600 [34].

While water consumption is preferred, for children who do not receive water, consuming alternative beverages may help to reduce the risk of dehydration. In the second stage of the analysis, we examined whether alternative beverages were consumed by those children who did not drink any water in the past 24 h. In the final stage of the analysis, we evaluated which children were at greatest risk of not drinking water using logistic regression models. This included a vector of potential household and environmental risk factors of receiving no water. One factor was type of water source, including dummy variables for four categories, as follows: piped, tanker, or bottled water (coded 1 if piped into dwelling, into yard, tanker truck, cart with small tank, and bottled water); well water (coded 1 if tube well or borehole, protected well, unprotected well); public tap; and river, spring, or rainwater (coded 1 if protected, unprotected spring, river, dam, lake, ponds, stream, canal, irrigation channel, rainwater, and other). We also evaluated WHO/UNICEF measure [35], of whether or not the household uses an improved water source (coded 1 if piped into dwelling, into yard, public tap, tube well or borehole, protected well, protected spring, rainwater; 0 if tanker truck, car with small tank, bottled water, unprotected well, unprotected spring, river, dam, lake, ponds, stream, canal, irrigation channel, rainwater, and other sources; WHO & UNICEF, 2013). We also expected children living in deprived households to be at greater risk. This was assessed using a dichotomous indicator of whether the household was deprived, based on a collapsed version of the standard DHS household wealth index available in the survey data (poorest and poorer were coded as deprived; middle wealth, richer, and richest were coded as not deprived). We also included place of residence (urban non-slum, urban slum, and rural).

Models evaluated potential disparities for age, gender, and social position, based on previous literature suggesting such inequalities in nutritional outcomes [36–38]. As a validity check, we included an indicator for whether the child had diarrhoea in the 2 weeks preceding the interview, as such a child should have been more likely to receive water. We also adjusted for maternal characteristics including maternal age, a categorical educational attainment measure (no schooling, primary school, secondary school, higher than secondary), religious affiliation of the household head (Hindu, Muslim, Christian, and other); caste (scheduled caste, scheduled tribe, other backwards class, and other/no caste). All models were estimated using STATAv12.1

## Results

### Prevalence of Children Not Receiving Water

Additional file 2 shows the prevalence rates of water consumption disaggregated by sex. Among children aged 6–59 months, who should receive water in addition to breast milk and complementary foods [39], we found that nearly one in ten (9.4 %, 95 % CI: 9.0 to 9.8 %) was reported by mothers to have consumed no water in the last 24 h. When scaled to India’s population, this equates to substantial numbers: Multiplying the proportion not receiving water by the UN estimates of the total population of 6–59 month olds for the year 2005 indicates that there were approximately 12,700,000 Indian children (95 % CI: 12,261,906 to 13,215,610) did not receive water in the last 24 h.

**Fig. 1:**
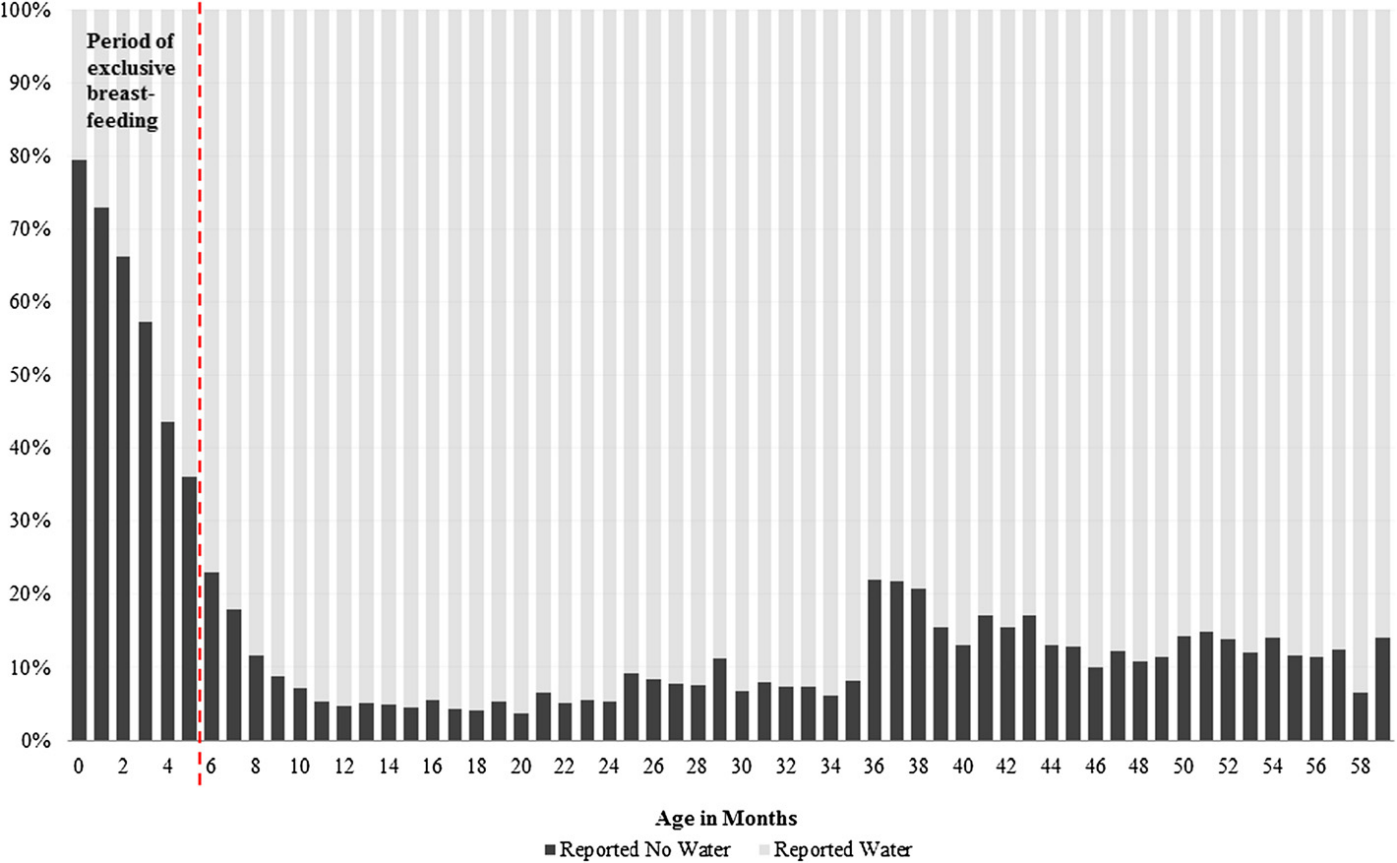
Percent receiving no water in the last 24 h by age, living children aged 0–59 months, NFHS-3

Figure 1 depicts the pattern of water consumption for each month of life. At 6 months nearly a quarter of children (22.9 %) did not receive water in the last 24 h. This proportion declines fairly steadily to its lowest point, 3.7 %, at 20 months of age. The proportion not receiving water increased further at 36 months of age, followed by slight declines for older ages.

### Alternative beverage consumption when water was not reported

**Fig. 2:**
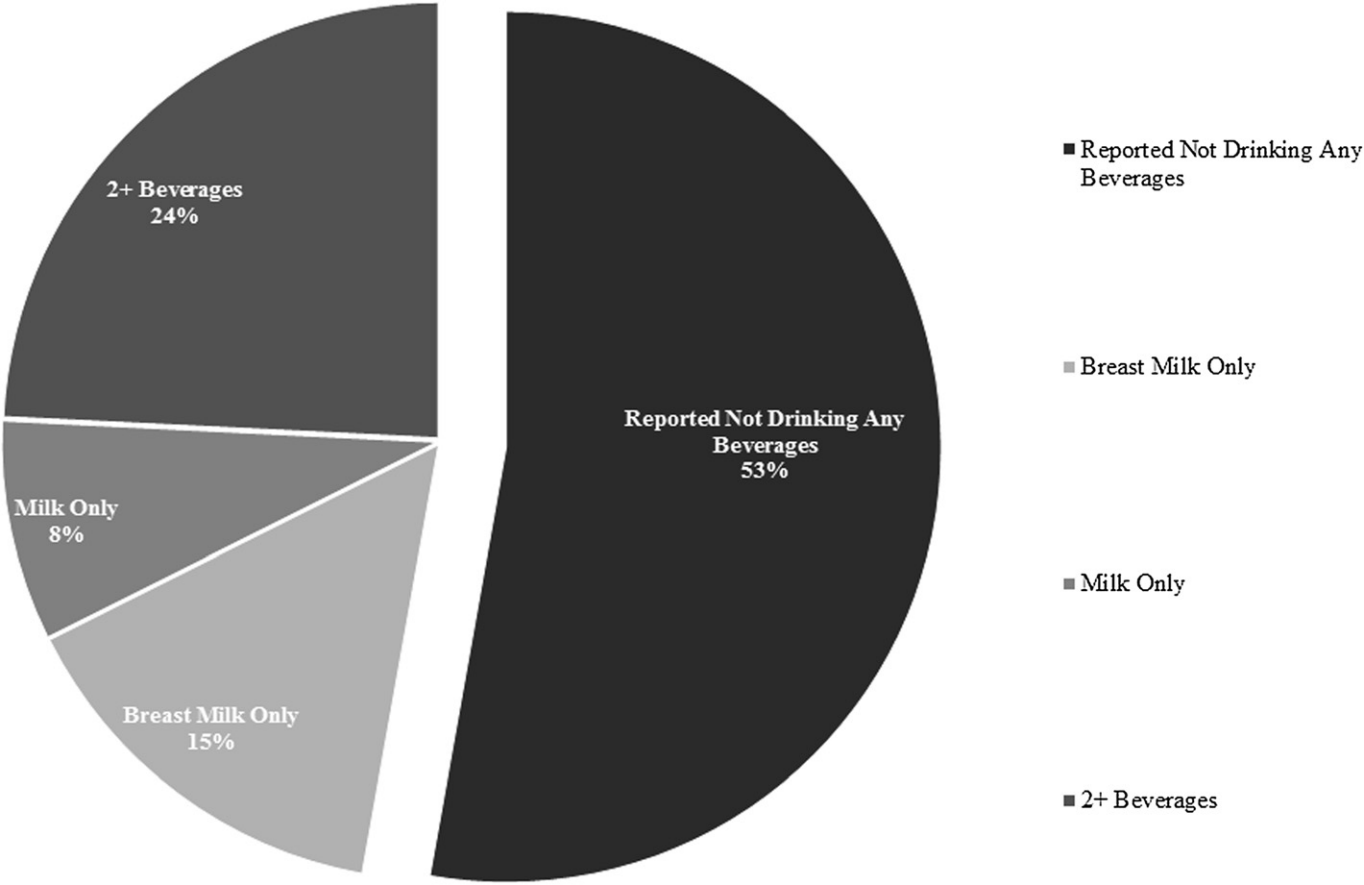
Beverage consumption among children whose mothers reported no water consumption in the last 24 h, living children aged 6–59 months, NFHS-3

We next asked, what do children drink instead of water? Figure 2 shows a pie chart of beverage consumption for the sub-sample of children who did not receive water in the past 24 h (*n* = 2,865). Among those children aged 6–59 months who received no water according to mothers’ reports, about half also received no other beverages at all (52.8 %), while nearly another quarter (23.0 %) received breast milk or fresh milk only. The remaining 24 % consumed either formula, “other liquid,” juice, or two or more beverages in the last 24 h. Less than 4 % of children who received no water had both fresh milk and breast milk in the last 24 h. 2.55 % received tea or coffee only, while just under 5 % received a combination of either tea/coffee and fresh milk (2.44 %) or tea/coffee and breast milk (2.37 %). Children over 2 years of age were more likely to consume non-milk beverages, including tea, coffee, and juice than those under 2 years (Additional file 3). For the sake of comparison, beverage consumption by age is also provided in Additional file 4 for children who did receive water in the last 24 h. The majority of children who received water additionally consumed some combination of (breast) milk, tea, and coffee.

### Which children do not receive water?

**Fig. 3:**
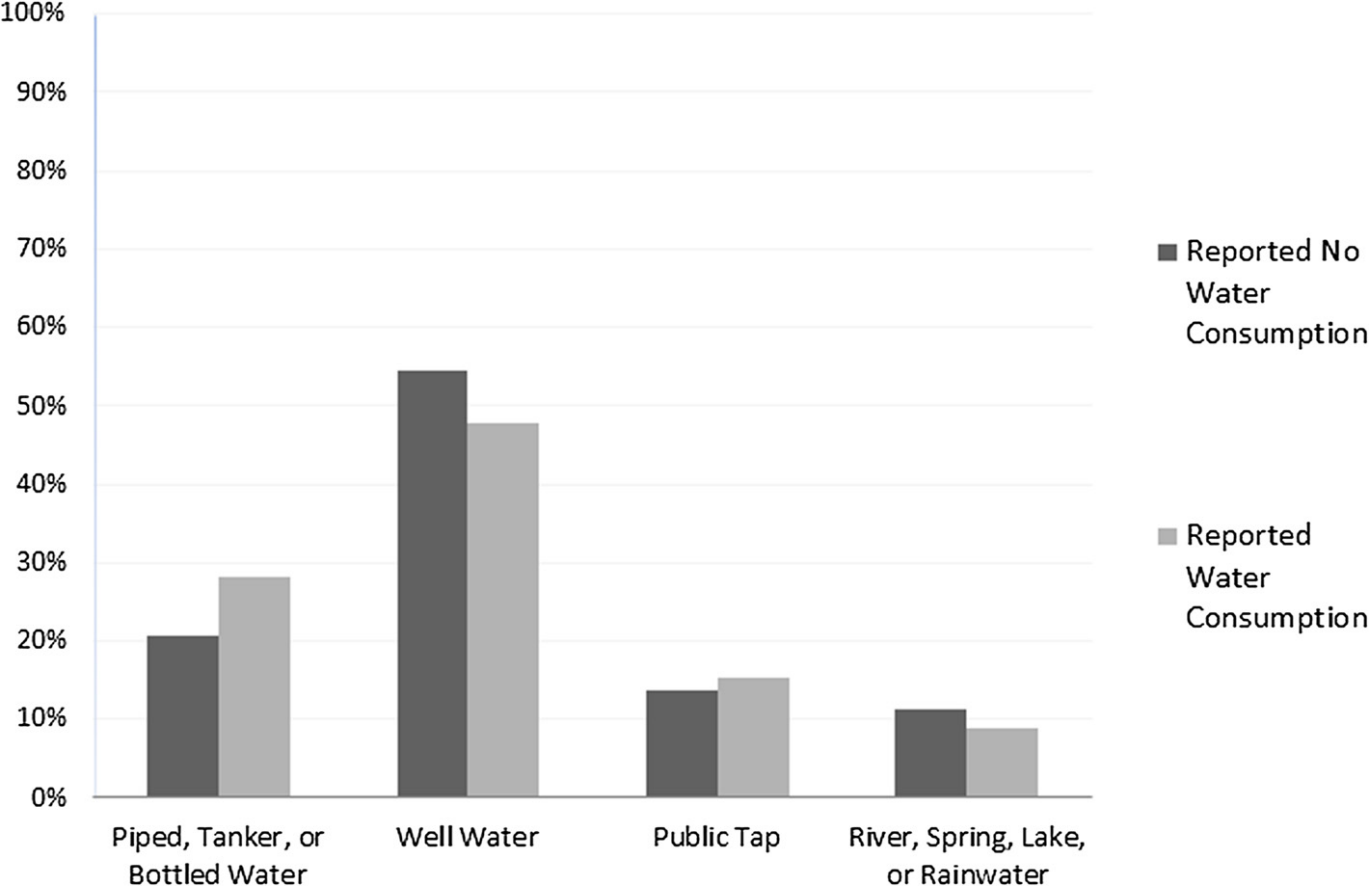
Household water source by child’s water consumption in the last 24 h, living children aged 6–59 months, NFHS-3

We anticipated that children living in households with unclean water and in more deprived settings would be less likely to receive water. We examined water consumption first by the four category measure of household water source, and second by the dichotomous WHO improved water source classification. Figure 3 shows a significant association between the four category measure of household water source and children’s water consumption (*χ*^2^ = 99.32; *p* < 0.001). The majority of children who did not consume any water in the last 24 h lived in households that used well-water as the main water supply (54 %). Higher risks were also seen in children whose households used river, spring, lake, or rainwater (11.2 % reported no water versus 8.74 % who reported having water). Conversely, a higher proportion of children who did consume water rely on piped, tanker, or bottled water (28.2 %) or a public tap (15.3 %) as compared to those who had no water (20.8 % and 13.7 % respectively). However, when we tested WHO/UNICEF coding of improved water sources, a chi-squared test (*χ*^2^ = 4.34; *p* = 0.04) did not identify discernible differences in children’s water consumption patterns (see Additional files 5 and 6).

Table 1 further evaluates the social patterning of the lack of water among Indian children using logistic regression models. Consistent with the above observations, we found that children living in households relying primarily on well water or river, spring, or rainwater as the main water source were significantly less likely (OR = 0.75; 95 % CI: 0.64 to 0.89 and OR = 0.70; 95 % CI: 0.53 to 0.92 respectively) to have received water than those in households with bottled, piped, or tanked water. Maternal education was positively associated with odds of receiving water; children whose mothers had a secondary (OR = 1.34; 95 % CI: 1.16 to 1.54) or higher than secondary educational level (OR = 1.56; 95 % CI: 1.13 to 2.15) had higher odds of receiving water compared to children whose mothers had no schooling. Children in deprived households were significantly less likely to receive water (OR = 0.84; 95 % CI: 0.74 to .096). There were no differences in the likelihood of water receipt by sex. Children aged 6–23 months had higher odds of receiving water (OR = 1.68; 95 % CI: 1.45 to 1.94) compared with children aged 48–59 months. As a further indication of validity of mothers’ report of giving water, we found that children who had diarrhoea in the last two weeks had significantly higher odds of receiving water (OR = 1.22; 95 % CI: 1.01 to 1.47).

**Table 1:** Fixed effects logistic regression models predicting whether child’s mother reported water consumption (yes = 1) in the last 24 h, living children 6–59 months, NFHS-3

	Child characteristics		Parental characteristics		HH characteristics		Water source		WHO/UNICEF water source	
	OR	95 % CI	OR	95 % CI	OR	95 % CI	OR	95 % CI	OR	95 % CI
Male	1.09	[0.98,1.20]	1.08	[0.98,1.20]	1.08	[0.97,1.19]	1.07	[0.97,1.19]	1.08	[0.97,1.19]
Child’s age in months										
48–59 months (ref)										
24–47 months	1.13	[0.99,1.29]	1.12	[0.98,1.29]	1.12	[0.98,1.28]	1.12	[0.97,1.28]	1.12	[0.98,1.28]
6–23 months	1.64***	[1.42,1.89]	1.68***	[1.45,1.94]	1.68***	[1.45,1.94]	1.68***	[1.45,1.94]	1.68***	[1.45,1.94]
Child had diarrhoea recently	1.20	[1.00,1.44]	1.22*	[1.01,1.46]	1.22*	[1.02,1.47]	1.22*	[1.01,1.47]	1.22*	[1.02,1.47]
Mother’s age in years			1.05***	[1.03,1.06]	1.05***	[1.03,1.06]	1.05***	[1.03,1.06]	1.05***	[1.03,1.06]
Mother’s education										
No schooling (ref)										
Primary school			1.02	[0.88,1.18]	0.96	[0.83,1.12]	0.95	[0.82,1.10]	0.96	[0.83,1.12]
Secondary school			1.57***	[1.39,1.79]	1.38***	[1.20,1.58]	1.34***	[1.16,1.54]	1.38***	[1.20,1.58]
Higher than secondary			2.01***	[1.48,2.72]	1.61**	[1.17,2.21]	1.56**	[1.13,2.15]	1.61**	[1.17,2.21]
Religion of HH head										
Hindu (ref)										
Muslim			0.85*	[0.73,0.98]	0.83*	[0.71,0.96]	0.84*	[0.72,0.98]	0.83*	[0.71,0.96]
Christian			0.86	[0.64,1.15]	0.82	[0.61,1.10]	0.84	[0.62,1.13]	0.82	[0.61,1.10]
Other religion			1.53*	[1.10,2.13]	1.47*	[1.06,2.04]	1.44*	[1.03,2.00]	1.47*	[1.06,2.04]
Caste										
No or other caste/tribe (ref)										
Scheduled caste			0.87	[0.74,1.02]	0.90	[0.76,1.05]	0.89	[0.76,1.05]	0.90	[0.76,1.05]
Scheduled tribe			0.77**	[0.64,0.92]	0.83	[0.69,1.01]	0.84	[0.69,1.01]	0.83	[0.69,1.01]
Other backward class			1.06	[0.93,1.22]	1.08	[0.94,1.24]	1.09	[0.95,1.25]	1.08	[0.94,1.24]
Place of residence										
Urban, non-slum (ref)										
Urban, slum					0.92	[0.68,1.23]	0.85	[0.63,1.15]	0.92	[0.68,1.23]
Rural					0.85*	[0.74,0.97]	0.93	[0.81,1.08]	0.85*	[0.74,0.97]
Deprived household					0.81***	[0.71,0.91]	0.84**	[0.74,0.96]	0.81***	[0.71,0.91]
Source of water for household										
Bottled, piped, or tanker water (ref)										
Well water							0.75***	[0.64,0.89]		
Public tap							0.99	[0.81,1.21]		
River, spring, or rain water							0.70*	[0.53,0.92]		
WHO/UNICEF improved water source										
Unimproved water source										
Improved water source									1.00	[0.87,1.15]
Mother’s ID (fixed effects)	1.00	[1.00,1.00]	1.00	[1.00,1.00]	1.00	[1.00,1.00]	1.00	[1.00,1.00]	1.00	[1.00,1.00]
Observations	29504		29504		29504		29504		29504	

^*^p<.05 ^**^p<.01 ^***^p<.001

## Discussion

Our findings estimate that about 13 million Indian children aged 6–59 months did not drink water in the past 24 h. Among those who did not have water, roughly one-quarter consumed liquids other than breast or fresh milk, including juice and tea/coffee. Of the children not receiving water, half appeared not to have consumed any other fluids during the preceding 24 h. It is quite possible that older children drank water or other beverages outside the home which would not have been observed by their mothers or that other household members gave drinks to these children. No significant gender disparities in water consumption were found. Household deprivation and water sources were significant predictors of children not drinking water in the past 24 h of mothers’ recall.

As with any survey-based study, our analysis has several important limitations. First, we rely on mothers’ reports of child water and beverage consumption; for children who are old enough to walk, and especially for those who are cared for by siblings, mothers may not be fully aware of all water and beverage consumption. The pattern of water consumption varies by age in Fig. 1, potentially reflecting not only best breastfeeding practices, but also the potential influence of multiple caregivers and autonomous feeding. However, the short recall period of 24 h also enhances the validity of the recall data. Second, these figures may underestimate the prevalence of limited water consumption because the most deprived social groups may also be subject to being undercounted in survey data. Another limitation of our study is that we can measure whether or not water was consumed in the last 24 h, but not volume consumed; it may be the case that children received some water, but still not an adequate amount, suggesting that our results may provide a conservative estimate of water consumption. A final concern is that mothers may intentionally misreport water consumption as a result of social desirability or recall bias. However, the short recall period, paired with the increased water consumption among children who recently had diarrhoea, provide evidence of the validity of maternal reports. Moreover, we would expect water consumption to be over-reported rather the underreported in this case, again pointing to the conservative nature of our estimates.

Overall, our findings suggest that more deprived children appear to face a greater risk of dehydration and its sequelae. Our evidence that household water source is a significant predictor of water consumption is consistent with the possibility that mothers may be withholding water from children out of concern for water safety. Interestingly, this pattern did not hold when water source was measured by WHO/UNICEF improved water source standards, suggesting that perceptions about safe sources of drinking water may not closely match objective external assessments of water safety. Current WHO/UNICEF measures of improved water sources may mask perceived problems with the water supply and its accessibility.

## Conclusions

Additional research is needed to understand how perceptions of safe water align with actual water safety. Given the importance of adequate hydration for health [13, 20, 21], these findings, if further validated, may have important implications for child health policy in India. Unclean water is hazardous, but so too may be the consequences of limiting water consumption and substitution of other beverages. Perversely, the focus on unclean water may expose children to greater risks of dehydration. Policymakers might rectify the situation not only by improving water infrastructure, but also better informing public awareness of safe-water sources and children’s hydration needs.

## Additional files

Additional file 1:
**Descriptive statistics for living children ages 6–59 months, NFHS-3.**
Additional file 2:
**Frequencies for whether child’s mother reported water consumption in the last 24 h, living children aged 6–59 months, NFHS-3.**
Additional file 3:
**Frequencies of whether child’s mother reported consumption of various beverages in the last 24 h, living children aged 6–59 who received no water in the last 24 h.**
Additional file 4:
**Frequencies of whether child consumed various beverages in addition to water in the last 24 h, living children aged 6–59 who received water in the last 24 h.**
Additional file 5:
**WHO/UNICEF household water source by child's water consumption in the last 24 h, living children aged 6–59 months, NFHS-3.**
Additional file 6:
**Cross-tabulation of water source and whether child’s mother reported water consumption in the last 24 h, living children ages 6–59 months, NFHS-3.**
