# Supplementary material for: What do Indian children drink when they do not receive water? Statistical analysis of water and alternative beverage consumption from the 2005–2006 Indian National Family Health Survey
Source: BMC Public Health. 2015 Jul 5;15:612. doi: 10.1186/s12889-015-1946-4 (PMC4491259; doi:10.1186/s12889-015-1946-4)
Supplement: Additional file 1: — Descriptive statistics for living children ages 6–59 months, NFHS-3. [file 12889_2015_1946_MOESM1_ESM.docx]

Additional File 1. Descriptive statistics for living children ages 6-59 months, NFHS-3

| Variable Name | | n | Mean/% | St. Dev |
| --- | --- | --- | --- | --- |
| Child Received No Water | | 30656 | 9.35% | (0.29) |
| Male | | 30656 | 51.2% | (0.50) |
| Child's Age (Months) | | 30656 | 27.9 | (14.69) |
| Child Had Diarrhoea Recently | | 30624 | 10.1% | (0.30) |
| Mother's Age (Years) | | 30656 | 26.4 | (5.18) |
| Mother's Education (Highest Level Completed) | | 30655 |  |  |
|  | *No Schooling* | 12737 | 41.5% | (0.49) |
|  | *Primary School* | 4327 | 14.1% | (0.35) |
|  | *Secondary School* | 11400 | 37.2% | (0.48) |
|  | *Higher than Secondary* | 2191 | 7.15% | (0.26) |
| Religious Affiliation (HH Head) | | 30650 |  |  |
|  | *Hindu* | 20451 | 66.7% | (0.47) |
|  | *Muslim* | 5430 | 17.7% | (0.38) |
|  | *Christian* | 3358 | 11.0% | (0.31) |
|  | *Other Religion* | 1411 | 4.60% | (0.21) |
| Caste | | 29387 |  |  |
|  | *Scheduled Caste* | 5447 | 18.5% | (0.39) |
|  | *Scheduled Tribe* | 5320 | 18.1% | (0.39) |
|  | *Other Backwards Class* | 9851 | 33.5% | (0.47) |
|  | *Other/No Caste* | 8769 | 29.8% | (0.46) |
| Deprived Household | | 30656 | 37.1% | (0.48) |
| Place of Residence | | 30656 |  |  |
|  | *Urban, Non-Slum* | 9350 | 30.5% | (0.46) |
|  | *Urban, Slum* | 2104 | 6.86% | (0.25) |
|  | *Rural* | 19202 | 62.6% | (0.48) |
| Main Water Source for HH | | 30648 |  |  |
|  | *Piped Water, Tanker, or Bottled Water* | 8404 | 27.4% | (0.45) |
|  | *Well Water* | 14855 | 48.5% | (0.50) |
|  | *Public Tap* | 4635 | 15.1% | (0.36) |
|  | *River, Spring, or Rainwater* | 2754 | 8.99% | (0.29) |
| WHO/UNICEF Improved Water Source | | 30648 |  |  |
|  | *Improved Water Source* | 25157 | 82.1% | (0.38) |
|  | *Unimproved Water Source* | 5491 | 17.9% | (0.38) |
