# Supplementary material for: What do Indian children drink when they do not receive water? Statistical analysis of water and alternative beverage consumption from the 2005–2006 Indian National Family Health Survey
Source: BMC Public Health. 2015 Jul 5;15:612. doi: 10.1186/s12889-015-1946-4 (PMC4491259; doi:10.1186/s12889-015-1946-4)
Supplement: Additional file 2: — Frequencies for whether child’s mother reported water consumption in the last 24 h, living children aged 6–59 months, NFHS-3. [file 12889_2015_1946_MOESM2_ESM.docx]

Additional File 2. Frequencies for whether child consumed water in the last 24 hours, living children aged 6-59 months, NFHS-3

|  | *Overall* | | *Males* | | *Females* | |
| --- | --- | --- | --- | --- | --- | --- |
|  | Freq. | % | Freq. | % | Freq. | % |
| Reported Water Consumed | 27,791 | 90.7 | 14,288 | 91.1 | 13,503 | 90.2 |
| Did Not Report Water Consumed | 2,865 | 9.4 | 1,393 | 8.9 | 1,472 | 9.8 |
| Total | 30,656 | 100 | 15,681 | 100 | 14,975 | 100 |
