# Supplementary material for: What do Indian children drink when they do not receive water? Statistical analysis of water and alternative beverage consumption from the 2005–2006 Indian National Family Health Survey
Source: BMC Public Health. 2015 Jul 5;15:612. doi: 10.1186/s12889-015-1946-4 (PMC4491259; doi:10.1186/s12889-015-1946-4)
Supplement: Additional file 3: — Frequencies of whether child’s mother reported consumption of various beverages in the last 24 h, living children aged 6–59 who received no water in the last 24 h. [file 12889_2015_1946_MOESM3_ESM.docx]

Additional File 3: Frequencies of whether child’s mother reported consumption of various beverages in the last 24 hours, living children aged 6-59 who received no water in the last 24 hours

|  | *All Children* | | *Children Aged 6-24 Months* | | *Children Aged 25-59 Months* | |
| --- | --- | --- | --- | --- | --- | --- |
|  | Freq. | % | Freq. | % | Freq. | % |
| Nothing To Drink | 1513 | 52.8 | 194 | 18.7 | 1,319 | 72.2 |
| Breast Milk Only | 424 | 14.8 | 410 | 39.5 | 14 | 0.77 |
| Milk Only | 234 | 8.17 | 45 | 4.34 | 189 | 10.3 |
| 3+ Beverages | 172 | 6.00 | 125 | 12.1 | 47 | 2.57 |
| Milk And Breast Milk | 108 | 3.77 | 102 | 9.84 | 6 | 0.33 |
| Tea/Coffee Only | 73 | 2.55 | 7 | 0.68 | 66 | 3.61 |
| Tea/Coffee And Milk | 70 | 2.44 | 15 | 1.45 | 53 | 2.90 |
| Tea/Coffee And Breast Milk | 68 | 2.37 | 59 | 5.69 | 11 | 0.60 |
| Other Liquid Only | 42 | 1.47 | 1 | 0.10 | 41 | 2.24 |
| Formula Only | 31 | 1.08 | 6 | 0.58 | 25 | 1.37 |
| Milk And Formula | 27 | 0.94 | 11 | 1.06 | 16 | 0.88 |
| Other Liquid And Breast Milk | 24 | 0.84 | 24 | 2.31 |  |  |
| Formula And Breast Milk | 21 | 0.73 | 20 | 1.93 | 1 | 0.05 |
| Milk And Other Liquid | 13 | 0.45 | 2 | 0.19 | 11 | 0.60 |
| Juice And Milk | 9 | 0.31 | 2 | 0.19 | 7 | 0.38 |
| Juice And Breast Milk | 9 | 0.31 | 9 | 0.87 |  |  |
| Juice Only | 8 | 0.28 | 1 | 0.10 | 7 | 0.38 |
| Tea/Coffee And Formula | 7 | 0.24 | 3 | 0.29 | 4 | 0.22 |
| Tea/Coffee And Other Liquid | 5 | 0.17 |  |  | 5 | 0.27 |
| Juice And Tea/Coffee | 3 | 0.10 |  |  | 3 | 0.16 |
| Juice And Formula | 3 | 0.10 | 1 | 0.10 | 2 | 0.11 |
| Formula And Other Liquid | 1 | 0.03 |  |  | 1 | 0.05 |
| Total | 2865 | 100 | 1,037 | 100 | 1,828 | 100 |
