# Supplementary material for: What do Indian children drink when they do not receive water? Statistical analysis of water and alternative beverage consumption from the 2005–2006 Indian National Family Health Survey
Source: BMC Public Health. 2015 Jul 5;15:612. doi: 10.1186/s12889-015-1946-4 (PMC4491259; doi:10.1186/s12889-015-1946-4)
Supplement: Additional file 4: — Frequencies of whether child consumed various beverages in addition to water in the last 24 h, living children aged 6–59 who received water in the last 24 h. [file 12889_2015_1946_MOESM4_ESM.docx]

Additional File 4: Frequencies of whether child consumed various beverages in addition to water in the last 24 hours, living children aged 6-59 who received water in the last 24 hours

|  | *All Children* | | *Children Aged 6-24 Months* | | *Children Aged 25-59 Months* | |
| --- | --- | --- | --- | --- | --- | --- |
|  | Freq. | % | Freq. | % | Freq. | % |
| Only Water to Drink | 2,808 | 13.80 | 297 | 3.46 | 2,511 | 21.31 |
| Water and Breast Milk | 3,033 | 14.90 | 2,600 | 30.33 | 433 | 3.68 |
| Water and Milk | 2,225 | 10.93 | 544 | 6.35 | 1,681 | 14.27 |
| Water, Milk, and Breast Milk | 1,930 | 9.48 | 1,633 | 19.05 | 297 | 2.52 |
| Water and Tea/Coffee | 2,004 | 9.85 | 210 | 2.45 | 1,794 | 15.23 |
| Water, Tea/Coffee, and Milk | 2,566 | 12.61 | 465 | 5.42 | 2,101 | 17.83 |
| Water, Tea/Coffee, and Breast Milk | 2,071 | 10.17 | 1,401 | 16.34 | 670 | 5.69 |
| Water and Other Liquid | 437 | 2.15 | 44 | 0.51 | 393 | 3.34 |
| Water and Formula | 233 | 1.14 | 54 | 0.63 | 179 | 1.52 |
| Water, Milk, and Formula | 363 | 1.78 | 129 | 1.50 | 234 | 1.99 |
| Water, Other Liquid, and Breast Milk | 511 | 2.51 | 432 | 5.04 | 79 | 0.67 |
| Water, Formula, and Breast Milk | 287 | 1.41 | 266 | 3.10 | 21 | 0.18 |
| Water, Milk And Other Liquid | 426 | 2.09 | 111 | 1.29 | 315 | 2.67 |
| Water, Juice, and Milk | 315 | 1.55 | 111 | 1.29 | 204 | 1.73 |
| Water, Juice, and Breast Milk | 154 | 0.76 | 121 | 1.41 | 33 | 0.28 |
| Water and Juice | 106 | 0.52 | 20 | 0.23 | 86 | 0.73 |
| Water, Tea/Coffee, and Formula | 135 | 0.66 | 36 | 0.42 | 99 | 0.84 |
| Water, Tea/Coffee, and Other Liquid | 558 | 2.74 | 58 | 0.68 | 500 | 4.24 |
| Water, Juice, and Tea/Coffee | 102 | 0.50 | 12 | 0.14 | 90 | 0.76 |
| Water, Juice, and Other Liquid | 34 | 0.17 | 7 | 0.08 | 27 | 0.23 |
| Water, Juice, and Formula | 23 | 0.11 | 11 | 0.13 | 12 | 0.10 |
| Water, Formula, and Other Liquid | 34 | 0.17 | 11 | 0.13 | 23 | 0.20 |
| Total | 20,355 | 100 | 8,573 | 100 | 11,782 | 100 |
