# Supplementary figures and images for: What do Indian children drink when they do not receive water? Statistical analysis of water and alternative beverage consumption from the 2005–2006 Indian National Family Health Survey
Source: BMC Public Health. 2015 Jul 5;15:612. doi: 10.1186/s12889-015-1946-4 (PMC4491259; doi:10.1186/s12889-015-1946-4)

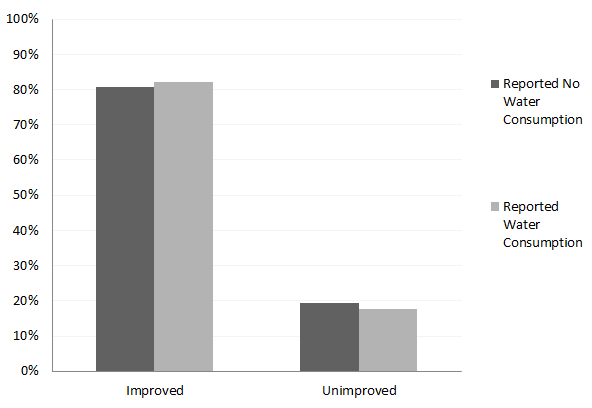

Supplement: Additional file 5: — WHO/UNICEF household water source by child's water consumption in the last 24 h, living children aged 6–59 months, NFHS-3. [file 12889_2015_1946_MOESM5_ESM.tiff]
