# Supplementary material for: What do Indian children drink when they do not receive water? Statistical analysis of water and alternative beverage consumption from the 2005–2006 Indian National Family Health Survey
Source: BMC Public Health. 2015 Jul 5;15:612. doi: 10.1186/s12889-015-1946-4 (PMC4491259; doi:10.1186/s12889-015-1946-4)
Supplement: Additional file 6: — Cross-tabulation of water source and whether child’s mother reported water consumption in the last 24 h, living children ages 6–59 months, NFHS-3. [file 12889_2015_1946_MOESM6_ESM.docx]

Additional File 6. Cross-tabulation of water source and whether child’s mother reported water consumption in the last 24 hours, living children ages 6-59 months, NFHS-3

|  | | Reported Water | % | Reported No Water | % | Total | % |
| --- | --- | --- | --- | --- | --- | --- | --- |
| *Water Source by Category* | |  |  |  |  |  |  |
|  | Piped, Tanker, or Bottled Water | 7,814 | 28.1 | 590 | 20.6 | 8,404 | 27.4 |
|  | Well Water | 13,296 | 47.9 | 1,559 | 54.4 | 14,855 | 48.5 |
|  | Public Tap | 4,242 | 15.3 | 393 | 13.7 | 4,635 | 15.1 |
|  | River, Spring, Lake, or Rainwater | 2,431 | 8.75 | 323 | 11.3 | 2,754 | 8.99 |
|  | Total | 27,783 | 100 | 2,865 | 100 | 30,648 | 100 |
| *WHO/UNICEF Improved Water Source* | |  |  |  |  |  |  |
|  | Improved | 22846 | 82.3 | 2,311 | 80.7 | 25,157 | 82.1 |
|  | Unimproved | 4937 | 17.8 | 554 | 19.3 | 5,491 | 17.9 |
|  | Total | 27783 | 100 | 2,865 | 100 | 30,648 | 100 |
